# Spatiotemporal discrimination in attractor networks with short-term synaptic plasticity

**DOI:** 10.1007/s10827-019-00717-5

**Published:** 2019-05-27

**Authors:** Benjamin Ballintyn, Benjamin Shlaer, Paul Miller

**Affiliations:** 1 Neuroscience Program, Brandeis University, Waltham, MA 02453 USA; 2 Department of Physics, University of Auckland, Auckland, New Zealand; 3 Department of Biology, Brandeis University, Waltham, MA 02453 USA; 4 Volen National Center for Complex Systems, MS013, Brandeis University, Waltham, MA 02453 USA

**Keywords:** Attractors, Synaptic depression, Sequence encoding, Decision-making

## Abstract

We demonstrate that a randomly connected attractor network with dynamic synapses can discriminate between similar sequences containing multiple stimuli suggesting such networks provide a general basis for neural computations in the brain. The network contains units representing assemblies of pools of neurons, with preferentially strong recurrent excitatory connections rendering each unit bi-stable. Weak interactions between units leads to a multiplicity of attractor states, within which information can persist beyond stimulus offset. When a new stimulus arrives, the prior state of the network impacts the encoding of the incoming information, with short-term synaptic depression ensuring an itinerancy between sets of active units. We assess the ability of such a network to encode the identity of sequences of stimuli, so as to provide a template for sequence recall, or decisions based on accumulation of evidence. Across a range of parameters, such networks produce the primacy (better final encoding of the earliest stimuli) and recency (better final encoding of the latest stimuli) observed in human recall data and can retain the information needed to make a binary choice based on total number of presentations of a specific stimulus. Similarities and differences in the final states of the network produced by different sequences lead to predictions of specific errors that could arise when an animal or human subject generalizes from training data, when the training data comprises a subset of the entire stimulus repertoire. We suggest that such networks can provide the general purpose computational engines needed for us to solve many cognitive tasks.

**Electronic supplementary material:**

The online version of this article (10.1007/s10827-019-00717-5) contains supplementary material, which is available to authorized users.

## Introduction

Life is a sequence of interconnected events, such that our optimal response to one event is highly impacted by preceding events. For example, our actions following the sound of a fire alarm should be very different if we had just received a message about a forthcoming alarm test, from our actions following the same sound if we had just seen smoke emanate from a nearby chemistry laboratory. Moreover, the impact of auditory stimuli, most clearly noticeable in music or speech, is significantly affected by the stimuli preceding it. A musical note rarely sounds “pleasant” or “unpleasant” alone, yet can do so when expectations are set by an ongoing melody. Our response to someone yelling the word “run” is very different if the word “run” is preceded by the two words “do not”. Therefore, a key aspect of cognition is to enable our response to any stimulus to depend upon the preceding pattern of inputs. Fundamental to such an ability is the need for the pattern of neural activity in the underlying circuits to depend not just on the current stimulus, but on the entire sequence. In this paper, we propose one general method, based on point-attractor states, that the brain might use for achieving such sequence-dependent activity.

### Circuits with multiple point-attractor states

Networks of recurrently connected units are capable of producing a diversity of distinct point-attractor states (Brunel and Nadal 1998). The component units of these networks can be single neurons, or groups of correlated, similarly responsive neurons. The stable activity states they produce are called ‘attractors’, because when a stimulus causes the network’s activity to become similar to, or ‘nearby’ the attractor, the internal dynamics of the network cause that activity to shift toward (or be ‘attracted to’) the attractor state. The states are point attractors, because they are each described by a single, distinct set of firing rates of all the units. The set of rates can be represented as a single point in the high-dimensional space where each axis corresponds to a single unit’s rate. Such attractor states can arise when the neurons in a network change their connections *via* Hebbian synaptic plasticity (Mongillo et al. 2003; Bourjaily and Miller 2011). In autoassociative networks, such as the Hopfield network (Hopfield 1982), such plasticity allows for the learning and long-term storage of input patterns, which can be reconstructed later from partial or corrupted versions of the prior stimuli. In this scheme, after learning, attractor states of the network represent learned representations of the presented stimuli.

Modeling studies have also shown that networks with point-attractor states can underlie short-term memory and decision-making (Brunel 2003; Marti et al. 2008; Miller and Katz 2010). *In vivo* experiments have provided support for these ideas (Fuster 1973; Goldman-Rakic 1995; Miller et al. 1996; Jones et al. 2007; Sadacca et al. 2016; Latimer et al. 2015; Miller 2016). In recurrent networks with strong self-excitatory connections, units are capable of maintaining their firing state long after the presentation of a stimulus. Such behavior is known as bistability since the neuron has two stable firing rates--quiescent and active-- in the absence of stimulus. In such a network, the activity or lack thereof within a particular group of cells forms a basis for short- term memory of prior inputs (Wang 2001).

Recurrent networks with random excitatory connections have been shown to have particularly useful properties (Rigotti et al. 2010a, b). Random network connections in a recurrent network endow the neurons with ‘mixed-selectivity’ for combinations of stimuli, a property of neurons observed *in vivo* (Rigotti et al. 2013; Rao et al. 1997) and important for solving linearly non-separable tasks (such as the Exclusive-Or) (Bourjaily and Miller 2011).

If the inputs to neurons in an attractor network are relatively strong compared to the recurrent feedback, then when the network receives a new input its activity will switch to the new attractor state corresponding to the new input. In such an event, information about the prior input is lost. Alternatively, if the input is relatively weak then it can be insufficient to drive the activity away from the prior attractor state, and only information about the initial stimulus is retained. We suggest that this parameter dependence, which leads to either recency (strongest memory for the last stimulus) or primacy (strongest memory for the first stimulus) might be the basis of these observed recall phenomena (Murdock 1962). Given individual human subjects exhibit both primacy and recency in a single task (Healey and Kahana 2014), we investigate whether both effects can arise in a single heterogeneous network.

Evidence for quasistable point attractors in neural circuits *in vivo* arises from the observation of rapid transitions between relatively stationary states of coordinated network activity. Such rapid transitions can occur in the absence of a stimulus, or when the presented stimulus is constant or gradually ramping. For example, Mattia et al. (2013) found that in a visuomotor decision-making task, multi-unit recordings in pre-motor cortex revealed that groups of neurons transition suddenly between distinct activity states, forming a series of network states which ultimately settle into a stereotyped network activity pattern that predicts future movement. Additional *in vivo* evidence comes from rat and mouse gustatory and auditory cortices (Miller and Katz 2010; Jones et al. 2007; Mazzucato et al. 2015; Bathellier et al. 2012). Both sensory cortices show series of abrupt transitions between discrete network states, whose timing varies from trial-to-trial, such that the transitions may be obscured by typical analyses with cross-trial averaging.

Evidence of point-attractors has also been observed to govern place field remapping in the hippocampus (Wills et al. 2005). Place cell populations responded to gradual changes in the environment with a rapid transition in network state, thought to represent environmental context. Taken together, these studies provide extensive evidence for point-attractor dynamics *in vivo* (Miller 2016).

### Sequence-dependent memory

In order for a network to possess sequence-dependent memory, it must exhibit both of the following characteristics. First, it must respond to an external stimulus in a manner that discriminates between all possible incoming stimuli. Second, it must do so while retaining information about the pre-stimulus state of the network. An added complication is that this behavior must succeed in the presence of realistic noise fluctuations.

As demonstrated previously (Miller 2013), the dynamics of synaptic depression can enable a randomly connected attractor network to respond to new stimuli while retaining information about the pre-stimulus state. Once a stimulus is removed, the stable state of the network (a fixed point) can depend both on the most recent stimulus and on the state of the network prior to that stimulus. Repeating the process in this manner with successive stimuli can lead to a network state that depends on the properties of an entire sequence of stimuli. We showed that these networks can encode in distinct states the amplitude, duration, and number of stimuli presented to the network in a temporal sequence. The distinct activity states made use of the high-dimensional space of neural firing rates (Fusi et al. 2016), so that the different stimulus properties are not combined together as they would be in standard models, such as neural integrators (Koulakov et al. 2002; Lee et al. 1997; Miller and Wang 2006; Oestreich et al. 2006; Simen et al. 2011), which have low dimensionality (Ganguli et al. 2008a, b). In the successful models of ours, each stimulus was encoded as an input of identical magnitude to all cells, with different stimuli distinguished by the duration and amplitude of that input. Here we extend this result to show that versions of the same network can encode temporal sequences of different stimuli, in which the stimuli are distinguished according to which cells they activate.

We perform two discrimination tasks with the network. In the first, we consider sequences comprised of different patterns of two distinct stimuli, separated by short pauses, with six stimuli combined into the sequence. This task is motivated by an experiment (Morcos and Harvey 2016) in which a mouse is trained to turn left or right at the junction of a T-shaped maze according to whether it sees more visual cues on its left or right side when approaching the junction. Such a task can be solved by a circuit which takes the difference between the two counters or integrators (one for the left, one for the right) requiring only a one-dimensional representation of stimulus history (a single scalar). However, the authors found that neural activity stepped through sequences of states that depended on the entire preceding sequence of stimuli. The observed dynamics is more suggestive of the distributed coding present in a high-dimensional representation of stimulus history. Such a high-dimensional representation arises in the randomly connected network of bistable units that we consider here.

The second task requires discrimination between sequences of seven distinct stimuli presented without repetition. We test the network’s ability to discriminate between different permutations of the seven stimuli, and to what extent the final state of the network could be said to encode each one of the presented stimuli, compared to stimuli that had not been presented. The work is motivated by experiments on free recall of lists of presented words. In those experiments, better recall of the first one or two words in the list (primacy) and/or better recall of the final one or two words in the list (recency) is typically found (Murdock 1962; Neath 2010; Tan and Ward 2000; Neath and Crowder 1996).

We will test the extent to which our network possesses primacy and/or recency by comparing how successfully the final state can be used to identify the first stimulus, or the last stimulus, compared to stimuli in the middle of the sequence. A network with strong self-excitation and strong cross-inhibition may, after encoding one stimulus, suppress any change in its activity when later stimuli are presented. Such a network would produce strong primacy, with its final state being very similar to its state following just the first stimulus. Conversely, a network dominated by the external input, rather than the internal feedback, is more likely to shift its activity entirely to match the latest input pattern. Such a network would exhibit recency, with its final activity most similar to the pattern produced by the latest input alone. Strong noise currents are likely to enhance recency, since they can eventually wash out information stored earlier in the network.

The transient weakening of self-interactions, due to synaptic depression, endows the network with a propensity for self-avoiding trajectories in the space of firing rates, a useful feature for discriminating temporal information about sequences of stimuli (Romani et al. 2013; Miller 2013). Since units that were recently on are less likely to become active while the network is evolving towards a fixed point, the network preferentially takes large steps in activity space following new stimuli. Large steps mean a large number of new fixed points are within range. This facilitates discrimination of temporal sequences of stimuli because confusion occurs whenever two distinct initial states can be brought to the same final state *via* presentation of identical stimuli.

## Methods

**Fig. 1:**
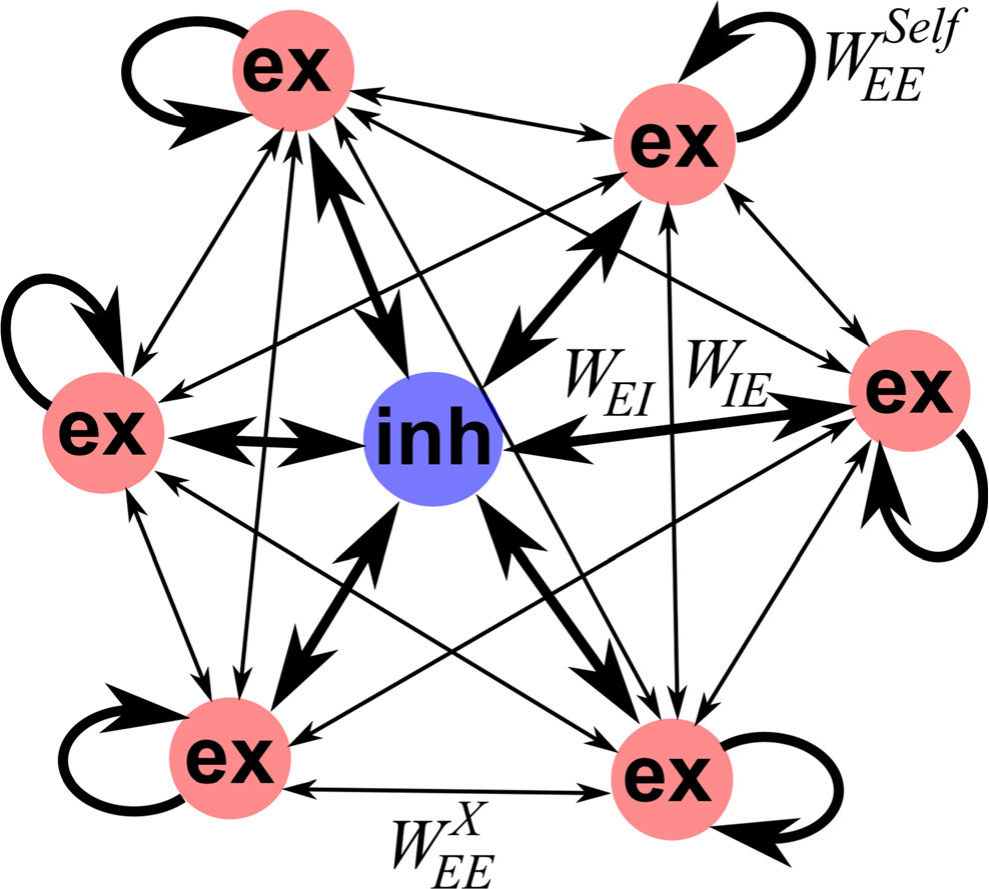
Schematic view of network. Excitatory self-connections render each excitatory unit bistable

We summarize the model and stimuli in Table 1 below. In short, we use firing-rate models of units (each representing a cluster of similarly responsive cells) with strong excitation within a unit and weaker, either excitatory or inhibitory, randomly assigned connections between units (Fig. 1). Connections are depressing. Distinct inputs of are of 250 ms duration, to distinct, potentially overlapping, subsets of units. We analyze the resulting network states after sequences of such inputs, with the goal of assessing the discriminability of diverse sequences.

**Table 1:** Model parameter ranges

Parameter type	Symbol	Values/Ranges
Time constants (*τ*)	*τ*_*r*_	100 ms
	${\tau}_{s_E}$	50 ms
	${\tau}_{s_I}$	5 ms
	*τ*_*D*_	500 ms
Sensitivity current (*Δ*)	*Δ*_*E*_	1
	*Δ*_*I*_	3
Thresholds (*Θ*)	*Θ*_*E*_	6
	*Θ*_*I*_	10, 12
Vesicle release probability (*p*_0_)	${p}_0^E$	1
	${p}_0^I$	.1
Maximum firing rates (*r*^*max*^)	${r}_E^{max}$	100 Hz
	${r}_I^{max}$	200 Hz
Max open receptor fraction ($\overset{\sim }{\alpha }$)	$\overset{\sim }{\alpha }$	1
Synaptic weights (*W*)	${W}_{EE}^{self}$	[71, 110]
	*σ*_*W*_	[0, 0.2]
	$$ {W}_{EE}^X $$	[0, .56]
	*W*_*EI*_	[.6, .72]
	*W*_*IE*_	[−580, −480]
	*W*_*II*_	0
Noise amplitude (*σ*)	*σ*	[.000, .0.002]
Stimulus fraction	*f*_*stim*_	[0, 1]
Stimulus Amplitude	*I*_*stim*_	[0.4, 1.8]
Stimulus Amplitude std. dev.	${\sigma}_{I_{stim}}$	[0, 0.1]*I_*stim*_
Stimulus duration	*T*_*stim*_	.25 s
Stimulus duration std. dev.	${\sigma}_{T_{stim}}$	[0, 0.1]*T_*stim*_
Starting fraction ON units	${f}_{start}^{on}$	[0, .2, .59]

All codes are available at https://github.com/primon23/Attractor-sequence-paper.

Summary of methods.

**A. Model Summary**

**Table:** 

Populations	N_E_ = 100 excitatory, N_I_ = 1 inhibitory
Connectivity	E-to-E: all-to-all with random strength, strong self-excitation
Neuron model	Firing rate model
Synapse model	Single exponential with synaptic depression
Plasticity	No long-term plasticity
Input	Sequences of spatially variable fixed duration square pulses
Measurements	Long lived (meta stable) firing rates following stimulus sequences

**B. Populations**

**Table:** 

**Name**	**Type**	**Number**
Excitatory (E)	Firing rate model	N_E_ = 100
Inhibitory (I)	Firing rate model	N_I_ = 1

**C. Connectivity**

**Table:** 

**Name**	**Source**	**Target**	**Distribution**
${W}_{EE}^{self}$	E	Self	Uniform *i.i.d* ∈ $\left[\left(1-{\sigma}_W\right),1\right]\ast {W}_{EE}^{self}$
${W}_{EE}^X$	E	Other E	Uniform *i.i.d* ∈ $\left[0,{W}_{EE}^{max}\right]$
*W*_*EI*_	E	I	Fixed at *W*_*EI*_ > 0
*W*_*IE*_	I	E	Fixed at *W*_*IE*_ < 0
*W*_*II*_	I	I	Fixed at 0

**D. Neuron and synapse model**

**Table:** 

**Name**	Firing rate model with synaptic depression
**Type**	Leaky integrate-and-fire with exponential conductance input
**Input current**	${I}_j(t)=\sum \limits_i{s}_i(t){W}_{ij}+{I}_j^{app}(t)+\sigma {\eta}_j(t)$
**Firing rate**	${\tau}_r\frac{d{r}_j(t)}{dt}=-{r}_j(t)+\frac{r^{max}}{1+{e}^{\left({\varTheta}_j-{I}_j(t)\right)/{\varDelta}_j}}$
**Synaptic gating variable**	${\tau}_{\boldsymbol{s}}\frac{d{s}_i(t)}{dt}=-{s}_{\boldsymbol{i}}(t)+\overset{\sim }{\alpha }{p}_0{r}_i(t){D}_i(t){\tau}_s\left(1-{s}_i(t)\right)$
**Depression Variable**	${\tau}_D\frac{d{D}_i(t)}{dt}=1-{D}_i(t)-{p}_0{r}_i(t){\tau}_D{D}_i(t)$

**E. Plasticity**

There is no long-term plasticity in this model, only short-term synaptic depression.

**F. Stimuli**

Stimuli are spatially variable synchronized square pulses of current (*I*_*stim*_) to a fraction of excitatory units (*f*_*stim*_). A summary of the stimulus sequences used for each task is given in the table below.

**Table:** 

**Task name**	**Sequence Length (** *L*_***seq***_ **)**	**# of distinct stimuli**	**# Sequences tested (** *N*_*seq*_ **)**	**Examples**
Nearest-Neighbor-Swap/Word Sequence task	7	7	7, 70	ABCDEFG, BACDEFG, *etc.*
2-choice task	6	2	64	LLLLLL, LRLRLR, *etc.*

**G. Measurements**

The primary measurement from which our analysis is derived is the high-dimensional state of the network following a given stimulus sequence. We measure the network state by taking the time-averaged firing rates of excitatory units following the final stimulus in a sequence.

1. **Firing rate model with synaptic depression**

**Fig. 2:**
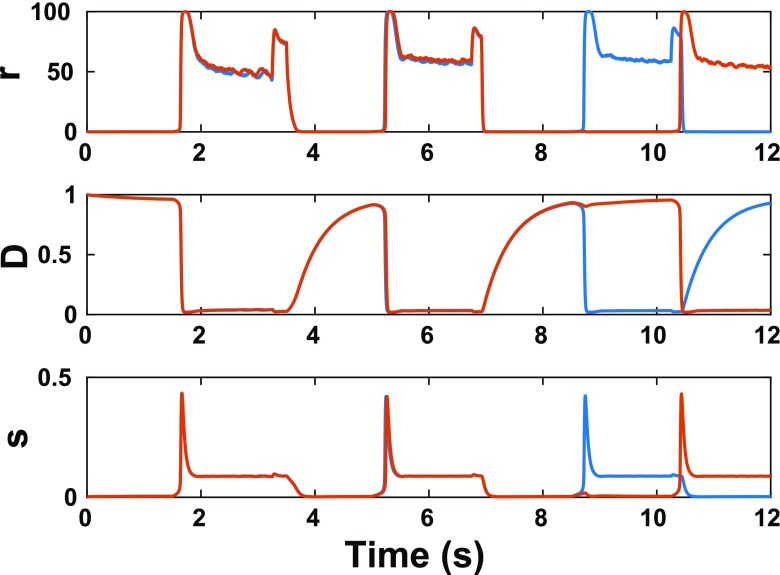
Example unit dynamics. Dynamic variables are plotted for 2 units in response to a sequence of 6 alternating stimuli. (**top**) Firing rates (***r***) (**middle**) depression variable (***D =*1**) indicates no depression) (**bottom**) synaptic output (***s***)

The basic unit of our network represents a pool of tightly coupled neurons. Their strong mutual interaction allows us to model this pool as a single unit, which can exhibit bistability with sufficient input current. The *i*^*th*^ unit is characterized by a single firing rate *r*_*i*_(*t*), dependent upon its net input current *via* a sigmoidal *f-*I curve,

1 $$ {\tau}_r\frac{d{r}_j(t)}{dt}=-{r}_j(t)+\frac{r^{max}}{1+{e}^{\left({\varTheta}_j-{I}_j(t)\right)/{\varDelta}_j}} $$

where *τ*_*r*_ = 10*ms* is the firing rate time constant, *Θ*_*i*_ is the threshold current, and *Δ*_*i*_ is the sensitivity current (controlling the slope of the *f-*I curve). The input current to the *i*^*th*^ cell-group is given by,

2 $$ {I}_j(t)={\sum}_i{s}_i(t){W}_{ij}+{I}_j^{app}(t)+\sigma {\eta}_j(t) $$

where *ση*_*j*_(*t*) is a white noise current with standard deviation *σ*_,_${I}_j^{app}(t)$ is the external applied current (stimulus), *s*_*i*_(*t*) is the dimensionless synaptic gating variable from the *i*^*th*^ group of cells, and *W*_*ij*_ is the connectivity matrix. The synaptic gating variable obeys

3 $$ {\tau}_{s_i}\frac{d{s}_i(t)}{dt}=-{s}_i(t)+\overset{\sim }{\alpha }{p}_0{r}_i(t){D}_i(t){\tau}_s\left(1-{s}_i(t)\right) $$

where the synaptic time constant *τ*_***s***_ describes the decay time of *s*_***i***_ following a significant decrease in firing rate. Excitatory units had a ${\tau}_{s_E}=50 ms$ while the single inhibitory unit had a ${\tau}_{s_I}=5 ms$. The increase in *s*_*i*_(*t*) toward unity is proportional to the firing rate *r*_*i*_(*t*) and the dimensionless depression variable *D*_*i*_(*t*). The fraction of open receptors in response to maximal vesicle release is $\overset{\sim }{\alpha }$ which we set to unity. The depression variable obeys,

4 $$ {\tau}_D\frac{d{D}_i(t)}{dt}=1-{D}_i(t)-{p}_0{r}_i(t){\tau}_D{D}_i(t) $$

where *τ*_*D*_ = 500*ms*. Example raw traces of the dynamical variables r, D, and s are shown for two units in Fig. 2 in response to a sequence of alternating stimuli. As external input causes a unit’s firing rate to increase, all of its outgoing synapses depress, reducing their synaptic output until reaching a steady state level.

The ranges of parameters used in our simulations are summarized in Table 1 below.

To assess the robustness of many of our results, we performed various parameter sweeps. These sweeps were over two-dimensional slices of both the four-dimensional space of network weight parameters, as well as the two-dimensional space spanned by the fraction of cells receiving input, and the amplitude of current making up the stimuli. The slices through parameter space intersect at the following fiducial set of parameter values, such that the non-varying parameters were held constant at these fiducial values:

$$ {\displaystyle \begin{array}{l}{W}_{EE}^X=.346,{W}_{EE}^{self}=89\\ {}{W}_{IE}=-540,{W}_{EI}=.665\\ {}{f}_{stim}=.59,{I}_{stim}=\mathrm{1.07.}\end{array}} $$

2) **Stimuli**

Each stimulus consists of a square current pulse of duration 250 ms to a randomly chosen fraction of excitatory units. We will describe these stimuli as spatial patterns, each consisting of *N*_*E*_ ∗ *f*_*stim*_ currents. Stimulus sequences consist of *L*_*seq*_ of these 250 ms current pulses delivered in some order separated by 1.5 s. This leaves a gap of 1250 ms during which the activity of the network may evolve without external stimulus toward a new fixed-point. The single inhibitory unit receives no external current.

3) **Measurements**

After a complete sequence of stimuli is presented, we wait 250 ms before beginning to average the firing rates. The average is taken over 1000 ms, *i.e.*, between 250 ms and 1250 ms after the final stimulus offset. This average firing rate is then used in all decoding efforts.

4) **Trials**

For each of the two discrimination tasks, we perform the following steps:

1. Select network parameters (5 connectivity matrix parameters, stimulus amplitude, stimulus fraction, stimulus variance, and noise level).
2. Generate 10 random instances of the network using these parameters
3. Generate the random input currents representing each stimulus sequence
4. Present the stimulus sequences to the networks and record the final network state after each sequence.
5. Evaluate the networks’ ability to discriminate between sequences and its ability to recall individual stimuli.

The random seed used for network and stimulus generation is distinct for each network, as is the random seed used to generate the noise within the units.

5) **Decoding discrimination**

We used two methods to assess how well the final state of activity in the network represented the distinct sequences of stimuli. In cases where we only needed to choose between two alternatives, we trained a perceptron. In the more frequent cases, where we wished to assess the number of individually distinct sequences represented and the similarity between the representations of different sequences, we performed analyses based on the confusion matrix. Both of these methods are described below.

### Confusion matrix

The ability of a network to discriminate between sequences depends on how often two distinct sequences lead to the same network activity. Due to the presence of noise, which broadens the distribution of activity states associated with each sequence, estimating this quantity is numerically intractable for all but the smallest networks. In cases where the number of distinct stimulus sequences is small, cluster analysis can be used. A more generally applicable method, which works both in large networks and for those receiving many stimuli, is to train a decoder with the mean “target” response to each stimulus sequence. Then any test response can be compared with the previously measured target responses to see which is closest.

**Fig. 3:**
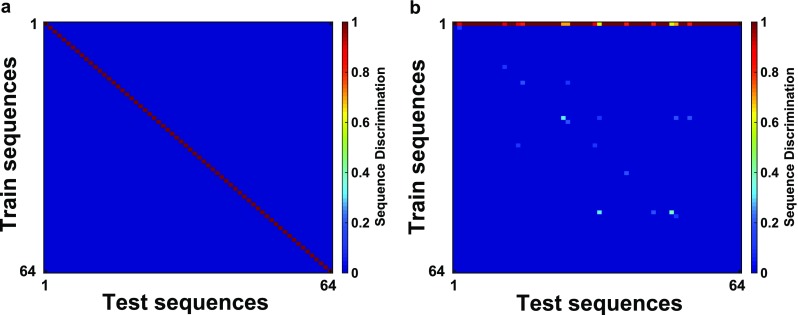
Confusion matrices for particular networks. Entry (***i***, ***j***) gives the fraction of times test sequence ***j*** was identified as target sequence ***i***. The particular network $\left({\boldsymbol{W}}_{\boldsymbol{EE}}^{\boldsymbol{self}}=\mathbf{88},\kern0.5em {\boldsymbol{W}}_{\boldsymbol{EE}}^{\boldsymbol{max}}=.\mathbf{476}\right)$ in (**a**) achieved perfect discrimination (***κ =*1**) while the network in (**b**) (${\boldsymbol{W}}_{\boldsymbol{EE}}^{\boldsymbol{self}}=\mathbf{80},{\boldsymbol{W}}_{\boldsymbol{EE}}^{\boldsymbol{max}}=.\mathbf{28}\Big)$ achieved a discrimination of ***κ = .*338**

Specifically, to quantify a network’s ability to discriminate between sequences, we first prepared the network in a quiescent initial state (although we have tested instances where the initial state contains a fraction of active excitatory units, see Supplementary Fig. 1). We then allowed the network to evolve with no external stimuli for at least 1.5 s, and used the resulting stable state as our initial condition for all subsequent stimulus presentations. We presented each stimulus sequence 10 times, recording the binarized final network states (where the mean firing rate of each unit in the post-sequence period is averaged and then subjected to a threshold of 30 Hz to label the unit either “on” or “off”) into a matrix ***X***_***train***_ to establish the supervised mean (“target”) response. We then presented each stimulus sequence 10 additional times (creating the test set ***X***_***test***_), and assigned to each a prediction based on the minimum L1-norm (shortest taxicab-distance) in state space (the *N*_*E*_ dimensional space where the activity of each unit has been labeled “on” or “off” using a threshold of 30 Hz) between this instance and the target response. Once we have determined the predicted sequence for each of the 10 repetitions of each of the *N*_*seq*_ sequences, we can construct a confusion matrix (Fig. 3), where element (*i*, *j*) represents the probability that actual presentation of a test sequence, *j*, results in the network predicting that the target sequence, *i*, was presented. Hence, each column of the confusion matrix sums to unity, and all of the information is contained in the off-diagonal elements, known as the error-rates.

### Perceptron training

A perceptron is a binary classifier, which essentially produces, *via* training, a plane through the high-dimensional space of the data (in this case the set of firing rates for 100 units represented in 100 dimensions). Data points are assigned to one output or another according to which side of the plane they reside.

For the left/right discrimination task, the perceptron was trained on a matrix of final network states (similar to ***X***_***train***_ but with the final states of sequences containing an equal number of both stimuli omitted). The perceptron was then evaluated on a test dataset ***X***_***test***_ and its accuracy (*a*_*decision*_ = fraction of correct choices) calculated.

6) **Sequence Discrimination ability**

We use a confusion matrix ***C*** to quantify the discrimination performance of the network. This matrix can be converted into several interesting scalar quantities. The total discrimination ability (*κ*) is defined as the fraction of times the network correctly classified the mean activity from a sequence, linearly rescaled such that a score of zero is obtained for discriminating no better than chance, and a score of unity is a perfect score:

$$ \kappa =1-\frac{\mathrm{mean}\ \mathrm{error}\ \mathrm{rate}}{1-\raisebox{1ex}{$1$}\!\left/ \!\raisebox{-1ex}{${N}_{seq}$}\right.} $$

where *N*_*seq*_ is the number of distinct sequences. Although it is possible for a network to perform worse than chance, we clip all *κ* values to be ∈ [0,1].

Note that a simple method for rescaling any performance metric of the confusion matrix *F*(*C*) is to use

$$ {F}_k\left(\boldsymbol{C}\right)=\frac{F\left(\boldsymbol{C}\right)-F\left({\boldsymbol{C}}_{chance}\right)}{F\left(\mathbf{1}\right)-F\left({\boldsymbol{C}}_{chance}\right)}\in \left[0,1\right] $$

where the identity matrix **1** is the error-less confusion matrix, and ***C***_*chance*_ is the uniform matrix with all entries equal to 1/*N*_*seq*_, representing the confusion matrix obtained by guessing uniformly.

7) **Left/Right sequence discrimination and evidence accumulation**

The first of the two discrimination tasks is based on the experiment of Morcos and Harvey (Morcos and Harvey 2016). The network is presented with a sequence of six cues of 2 types (modeling visual stimuli in the left or right visual field) and must make a binary decision based on the number of each type of stimuli (choose left if there are more left stimuli and right if there are more right stimuli). Hence, *N*_*types*_ = 2, *N*_*stim*_ = 6, *N*_*seq*_ = 2^6^ = 64. The network must keep track of how many of each stimulus type there is in the sequence and make a decision (turn left or right). In order to model the binary decision in the task we trained a perceptron to make the correct binary decision given a stimulus sequence (*e.g.* if a sequence contained more of stimulus type 1 than 2 the perceptron should output 0, otherwise if there were more of stimulus type 2 it should output 1).

8) **Seven-word list discrimination**

The second of the two discrimination tasks models word sequence recall in humans. The task consists of presenting sequences of 7 distinct stimuli and testing whether the sequences can be discriminated as well as whether individual stimuli can be recalled based on the final state of the network. Since there are 7 !  = 5040 possible input sequences, we sample this space using the Latin-squares method such that each stimulus type is presented the same number of times in each serial position across the whole set of sequences. Using this method we generate a list of 70 distinct 7-word sequences.

9) **Constituent stimulus recognition and probability of first recall**

To test which word in a sequence is most likely to be recalled first, we compared the state of the network after presentation of a complete sequence of stimuli to its state after presentation of an individual stimulus on its own. In this test we altered the length of lists, varying the number of stimuli from *N*_*stims*_ = 2 to *N*_*stims*_ = 10, in order to assess whether our networks produced the observed shift from primacy in short lists to recency in long lists.

10) **Primacy and recency**

To quantify the effects of primacy and recency in each network. In the first, we train perceptrons using ***X***_***train***_ to indicate, based on the final network state, whether stimulus type X was present in serial position *sp* of each sequence. Because there are 7 different stimulus types and 7 stimuli in each sequence, we train 7 perceptrons for each serial position (1 perceptron per stimulus type per serial position) for a total of 49 perceptrons. Then, for each serial position, we can compute 7 accuracies (1 for each stimulus type) and we set *a*_*sp*_ to be the mean of these 7 accuracies.

When “recall” ability (i. e., *a*_*sp*_) is plotted as a function of serial position, a primacy effect would show up on this plot as a higher “recall” ability for the earliest stimuli, while recency would show up as a higher “recall” ability for the latest stimuli. We calculate the primacy and recency scores for the 7-word list as the amount by which the scores for the first and final stimulus, respectively, are greater than the score for the middle (the 4th) stimulus.

## Results

### Left-right evidence accumulation and discrimination

**Fig. 4:**
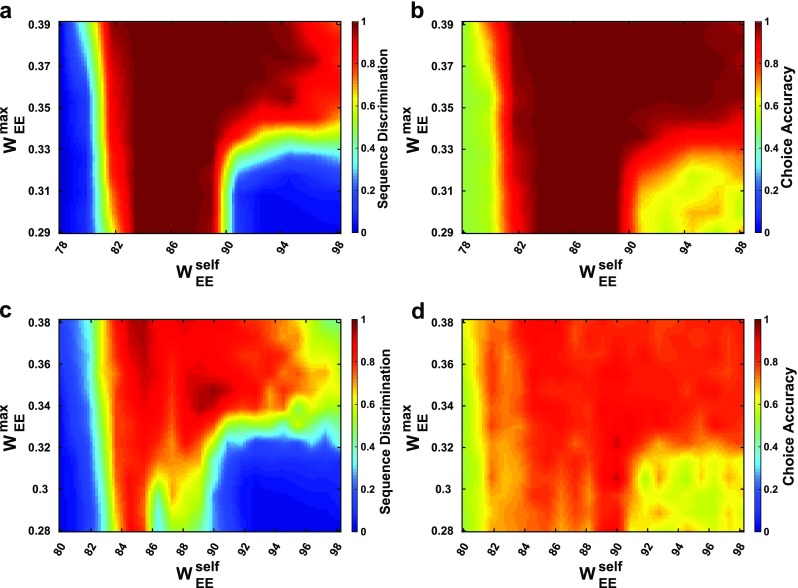
**a–b** Discrimination ability ***κ*** (**a**) and 2-choice accuracy (**b**) as a function of self and cross-excitation weights. One hundred twenty-one evenly spaced pairs of parameters were sampled and the results are interpolated across the self/cross-excitation space. All networks were noiseless with ***σ =*0*.*c–d** Same as a-b but for networks with a noise value of ***σ = .*002**. Two hundred seventy-three evenly spaced pairs of parameters were sampled

Our first goal was to assess whether the network could, following a sequence of six stimuli, each of which corresponded to either “left” or “right”, produce neural activity capable of distinguishing sequences with more left-stimuli from those with more right-stimuli. Our assessment comprised two tests. In the first test, we produced a confusion matrix, which indicates how distinct are the final activity states following each of the 64 possible combinations of six left/right stimuli. If all 64 stimulus patterns could be encoded distinctly in the network, we hypothesized that appropriate responses to those final activity states could be trained. As seen in Fig. 3, perfect discriminability between final states is possible for some networks. Moreover, for a broad range of parameters, the networks achieve good performance in this test (Fig. 4a).

For the second test, we trained an output (using a perceptron, see Materials and Methods) to produce a left or right response according to the greater number of left or right stimuli. In this manner, we could compare the response generated by the network with animal behavior. The results in Fig. 4b indicate again that good performance is achieved over a broad range of parameters.

A particular question when assessing whether such networks could be operating in the brains of animals is whether they are robust to the levels of firing rate variability typically observed *in vivo*. Moreover, as performance degrades in these networks – as it must with increased noise – we wished to assess whether the patterns of errors would allow us to make predictions about errors made by animals in behavioral tasks. Therefore, we tested networks with increased noise amplitude, *σ*. We find that for noise amplitude *σ* = .002 (corresponding to ~2-3 Hz noise oscillations) a range of networks are able to discriminate with >73% accuracy (Fig. 4c-d). The level of 73% is chosen because that is the accuracy that can be obtained by making choices according to the final stimulus alone, *i.e.*, without multi-item memory. We include networks without perfect performance in the following assessments, because the pattern of errors provides useful information about network function, which can be compared with *in vivo* behavior.

**Fig. 5:**
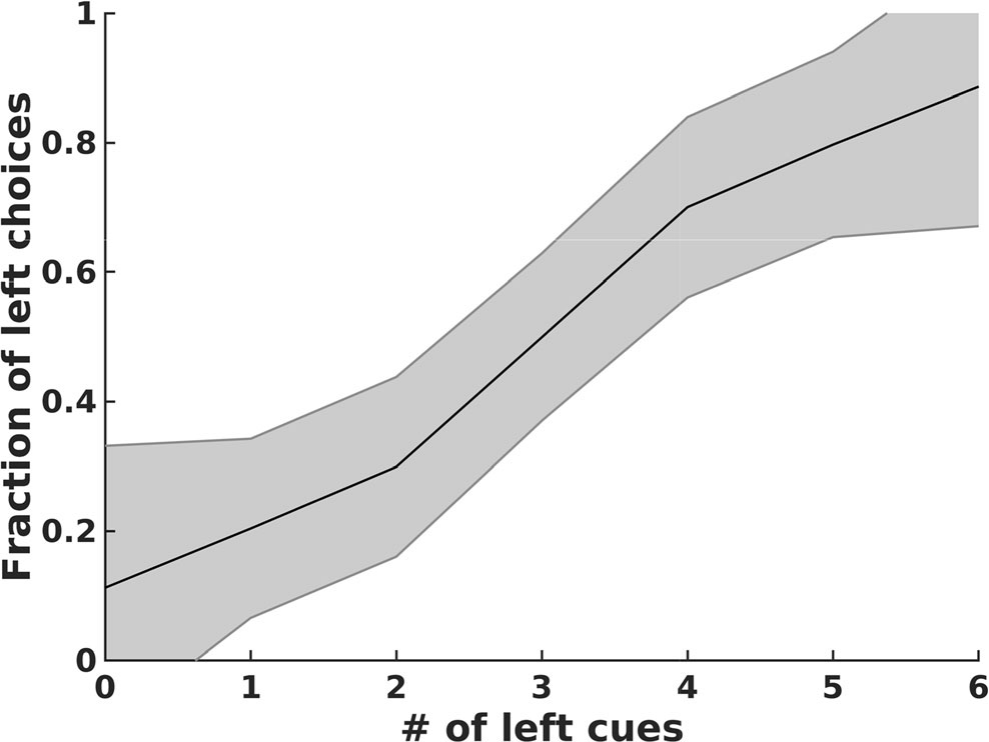
Likelihood of left choice *vs.* number of left cues. Psychometric curve for networks with a 2-choice accuracy > .73 (a threshold which could be achieved by guessing based solely on the last stimulus). Black line shows the mean left choice probability across networks while the shaded region shows the standard deviation

First, we measured how the probability of a left response depended on the number of left stimuli. We observe a sigmoidal psychometric curve, with probability of a correct choice increasing as the evidence in favor of that choice increases (Fig. 5). Such a result is in line with the observed animal behavior, and could be an indication of evidence accumulation, but also could be due to simpler effects such as making choices based on only the last (or the first) stimulus. For example, a network responding only to the final stimulus would be correct all of the time when all stimuli are identical, on 5/6 of the patterns with a 5-1 set of stimuli and 2/3 of the time with a 4-2 set of stimuli. Therefore, we investigated the patterns of errors and the extent to which final neural activity correlated with the total number of stimuli and with the final stimulus alone.

**Fig. 6:**
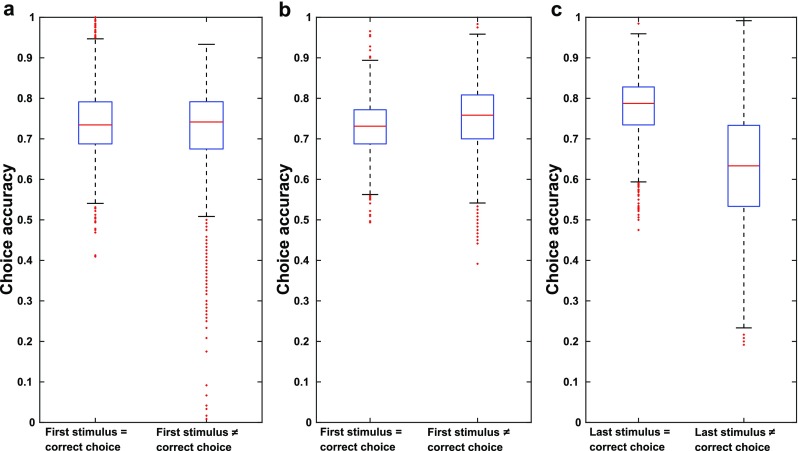
Patterns of choice errors relative to the first, 3rd, or last stimulus. Data shown is from all networks in Fig. 4c-d whose total choice accuracy was > .73. For all boxplots, red lines indicate the median, blue boxes indicate the range from 25th to 75th percentile, whiskers extend to 3 Median absolute deviations (MADs), and red “+” signs mark outliers (defined to be points beyond 3 MADs from the median. **a** Choice accuracies for sequences where the first stimulus does (left) or does not (right) support the correct choice. Mean choice accuracy for sequences where the first stimulus supported the correct choice were significantly higher than for sequences where the first stimulus did not support the correct choice (two-sample t-test: ***p ≪ .*001**). **b** Choice accuracies for sequences where the 3rd stimulus does (left) or does not (right) support the correct choice. Mean choice accuracy for sequences where the 3rd stimulus supported the correct choice were significantly lower than for sequences where the 3rd stimulus did not support the correct choice (two-sample t-test: ***p ≪ .*001**)***.*c** Choice accuracies for sequences where the last stimulus does (left) or does not (right) support the correct choice. Mean choice accuracy for sequences where the last stimulus supported the correct choice were significantly higher than for sequences where the first stimulus did not support the correct choice (two-sample t-test: ***p ≪ .*001**)

Indeed, as shown in Fig. 6, errors were significantly more likely when either the first or final stimulus supported the wrong choice. This is not simply because these scenarios correlate with less accumulated evidence, because when we separated trials according to whether an intermediate stimulus (stimulus 3 was used in Fig. 6b) supported the correct choice, a small, but oppositely directed effect was seen. Indeed, the small, opposite effect of the alignment of intermediate stimuli with correct choice is further evidence for the positive effect of alignment of first and last stimuli with correct choice. This is because on trials when a particular intermediate stimulus does not support the correct choice, it is more likely that the first and last stimulus do support the correct choice.

**Fig. 7:**
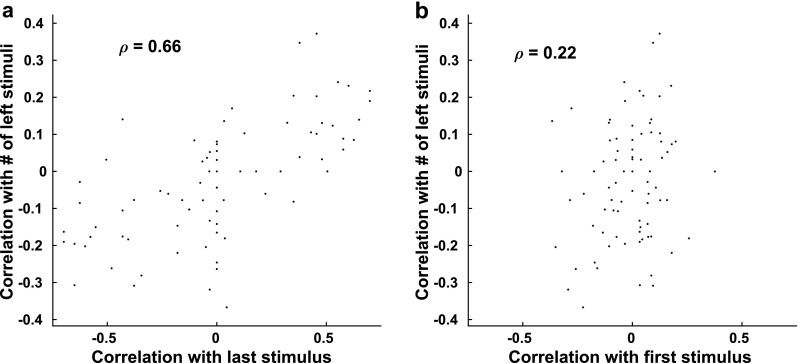
Correlations between excitatory unit activity and sequence properties from a network with perfect discrimination. **a** Correlations of unit activity with the last stimulus in a sequence were positively and significantly correlated with correlations of unit activity to the number of “left” stimuli (Pearson’s correlation ***ρ = .*66**, ***p ≪ .*001**). **b** Correlations of unit activity with the first stimulus in a sequence were not significantly correlated with correlations of unit activity to the number of “left” stimuli (Pearson’s correlation ***ρ = .*22**, ***p = .*052**)

To assess the neural underpinnings of the behavior, we measured, in a perfectly performing network, the correlation between each neuron’s final activity state with the total number of each stimulus (measuring the net evidence in favor of a particular choice), with the first stimulus, and with the final stimulus. Since the network produces perfect discrimination, all information about the sequence of stimuli is available across the population of neurons. However, in Fig. 7, we see that correlations were stronger for the final stimulus than either for net evidence or for the first stimulus and indeed those neurons with the most correlation with the net evidence were also most correlated with the final stimulus. Therefore, any measure of net evidence for a particular choice obtained from the final state of activity in the network is strongly impacted by the final stimulus type.

**Fig. 8:**
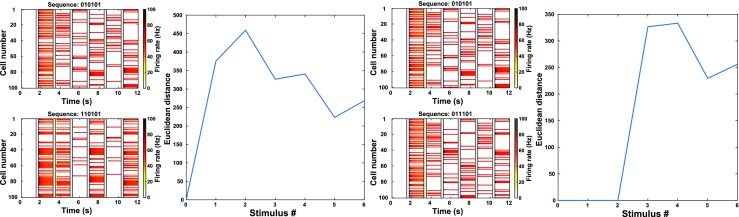
Comparisons of trajectories through activity space due to sequences that differ in only 1 position. Transparent gray bars mark periods when the stimulus is active. **a** Networks differ in their 1st stimulus. Left: Network activity *versus* time for each sequence. Each row indicates the firing rate of one unit. While successive stimuli cause changes in the activity patterns, those changes are in part determined by the prior activity pattern. Right: Euclidean distance between the network states induced by the two sequences. Since the 2 sequences differ in their first stimulus, the activity trajectories separate immediately. **b** Same as (**a**) except the two sequences compared here differ in their 3rd stimulus

It is revealing to visualize the trajectory of neural activity for two similar sequences by projecting to the space of the first three principal components. That is, we chose as a basis the three axes along which arose the greatest variation of neural activity across the sequences. In Fig. 8 (and see Supplementary Movies 1 and 2) we see that the two trajectories separate and move to distinct attractor states when the stimuli differ at a single point in the sequence. Thereafter, with identical following stimuli, the trajectories remain almost parallel, with a slow decline in the separation between activity states as further identical stimuli are added.

#### Comparison with animal studies

As found in the real mice (Morcos and Harvey 2016), network activity did not seem to directly represent different accumulated evidence values, but rather distinct network states dependent on the cue history. In our simulations, the network was started from the same initial conditions each trial. As such, for low noise values, identical sequences lead to very similar final network states (as can be seen in the confusion matrix). In the real mice however, it was found that identical stimulus sequences could lead to a diversity of network states. This may be due to multiple factors: the mouse’s starting internal state, at the time of stimulus onset, likely varies across trials, and there is likely trial to trial variability in the duration of visual stimuli or the intervals between stimuli. Therefore, we assessed the performance of our network with these real-world variabilities included.

To test how variability in the stimulus amplitudes and durations impacted sequence discrimination and choice accuracy, we simulated networks where either the stimulus amplitudes or durations were chosen by sampling from a Gaussian distribution where the standard deviation was 10% of the mean. The summary results from these tests are shown in Supplementary Figure 2. Allowing this much variability in the amplitude severely limited the networks’ ability to discriminate sequences (even with no external noise) although most networks still had *κ* values well above chance levels. Variability in the duration of stimuli also limited sequence discrimination compared to networks with constant duration although many networks were still able to achieve *κ* > .6.

In a third test, we trained the decoder/decision-network using states obtained from sequences with a 1500 ms inter-stimulus interval while testing on states obtained from sequences with a 3000 ms inter-stimulus interval (Supplementary Figure 3). Such an extension of the inter-stimulus interval has no detrimental impact on performance, indicating that the network has reached a stable steady state within 1500 ms following stimulus offset. Therefore, in the absence of a sufficiently strong noise fluctuation, the stable state persists no matter the duration of the inter-stimulus interval and the next stimulus impacts the network’s activity in the same way that it would, given a shorter interval.

In a fourth test, perceptrons were trained on states produced by stimulus sequences with a stimulus amplitude of either *I*_*stim*_ or *I*_*stim*_ + .1 ∗ *I*_*stim*_. That is, all stimuli in a particular trial had the same amplitude which could take on one of these two values. Doing this allowed the readout network to produce perfect choice accuracies across a range of parameters (Supplementary Figure 4).

Finally, to determine if the network’s performance was dependent on an initially quiescent network state (a strong assumption for any brain network) we simulated 4 different cases whose summary results may be seen in Supplementary Figure 1. In the first case, we initialized the network to have 59% $\left({f}_{start}^{on}=.59\right)$ of its units on at the start of every trial (the same units were set to be on across trials and sequences) as well as 59% (*f*_*stim*_ = .59) of the units receiving external input from each stimulus. The other 3 cases covered the following parameter pairs: 1) $\left({f}_{start}^{on}=.59,{f}_{stim}=.2\right)$ 2) $\left({f}_{start}^{on}=.2,{f}_{stim}=.59\right)$ 3) $\left({f}_{start}^{on}=.2,{f}_{stim}=.2\right)$. These results show that a quiescent initial state is not necessary for perfect sequence discrimination (perfect discrimination was achieved for broad ranges of parameters when *f*_*stim*_ = .59, Supplementary Figure 1 a-b). This suggests that an initial quiescent state is not necessary for the sequence discrimination properties of the network. However, when *f*_*stim*_ is reduced to .2 (Supplementary Figure 1 c-d), sequence discrimination is reduced across almost all of the parameters tested. This suggests that the initial, baseline, state of the network has a small but noticeable effect on the discrimination performance while the fraction of units receiving stimuli has a much larger impact on performance. However, it is necessary for the initial state to be the same across all presentations of a particular sequence if all presentations are to lead to the same final state. It may be possible to map activity states produced by identical sequences with differing starting states to a common behavioral output, although this was not attempted in our simulations.

#### Non-repeating sequences of 7 stimuli

The second task we performed is analogous to word recall in humans. We presented 70 different sequences (each with 7 distinct stimuli) to the network and again asked how well the network could discriminate between each sequence as well as whether the location and identity of a given stimulus could be identified solely by the final network state (recall). As in the two-item task, we observed trajectories through activity space that diverged upon a change in stimulus and remained divergent as successive stimuli were presented (Supplementary Movies 3-4). We found again that a wide range of networks could perfectly discriminate between all 70 sequences (Supplementary Figure 5).

#### Primacy and Recency

**Fig. 9:**
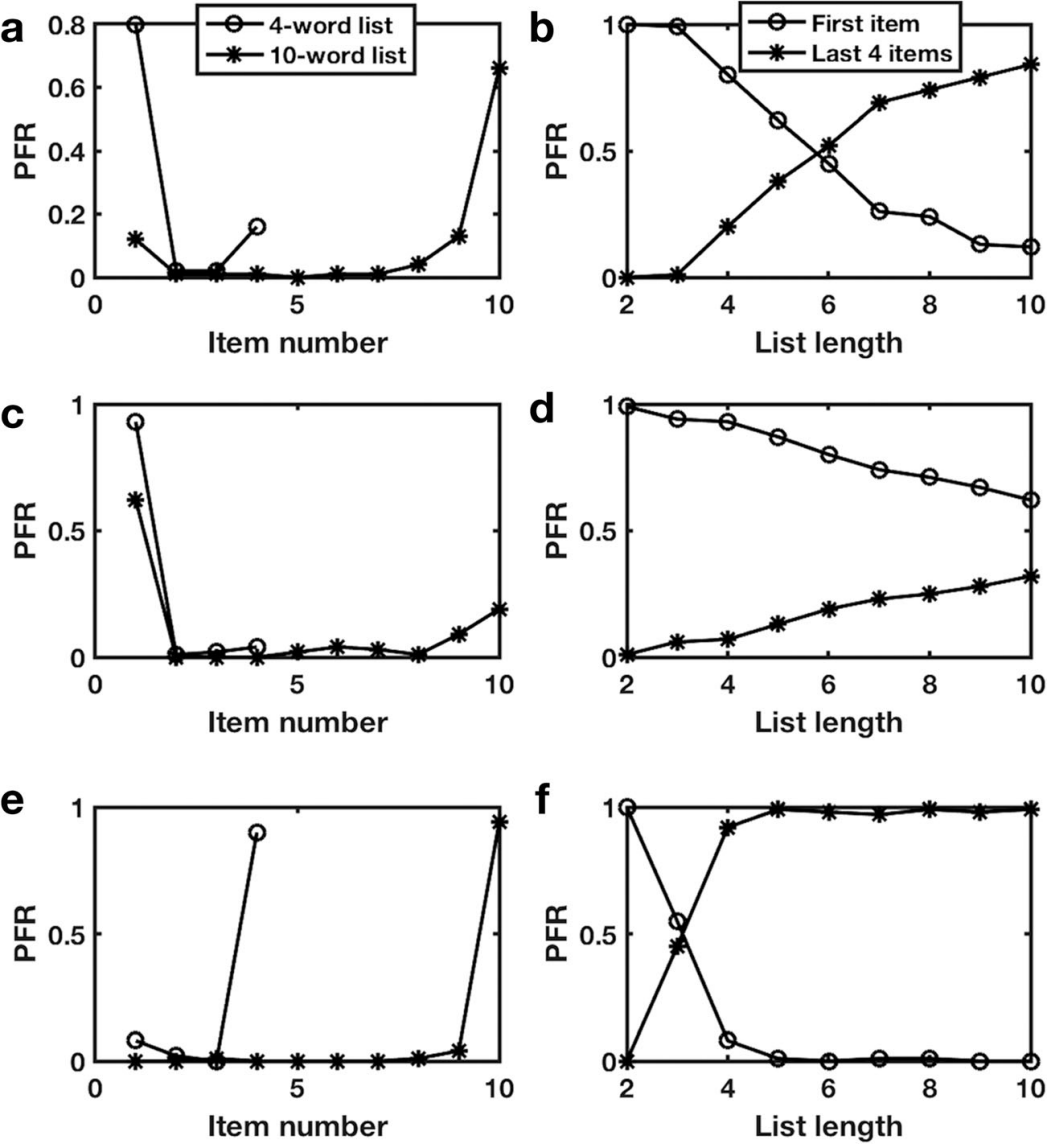
Probability of first recall exhibits primacy in short multi-item lists and recency in long multi-item lists. **a**, **b**: With appropriate parameters (***I***_***stim***_***=*0.9*±*0.2**) matches behavioral data. **c**, **d:** Reduced stimulus strength (***I***_***stim***_***=*0.85*±*0.2**) primacy dominates, but still decreases with increased list length. **e**, **f:** Increased stimulus strength (***I***_***stim***_***=*1.0*±*0.2**) recency dominates in all lists beyond length 3. **a**, **c**, **e:** Probability of first recall (PFR) as a function of item position in the list for 4-item lists (circles) or 10-item lists (asterisks). **b**, **d**, **f:** Probability of recall for the first item (circles) or up to the last 4 items excluding the first item (asterisks) as a function of list length

In the 7-word task, the network state at the end of the stimulus sequence determines which of the stimuli is first recalled. Therefore, we compared the final network activity pattern to the patterns produced by each of the stimuli alone and took the most similar individual-stimulus pattern to correspond to the stimulus first recalled. We found significant impacts of stimulus strength and strength of internal network connections. If the stimuli are relatively stronger and the network’s internal connections are relatively weaker, then the network’s activity pattern follows that of later stimuli (more recency) whereas in the opposite condition, more primacy is observed. For a very narrow range of intermediate stimulus strengths networks exhibited both primacy and recency in first recall. When we allowed stimulus strength to vary randomly about a mean along a sequence, the intermediate range of both primacy and recency broadened. Moreover, ubiquitously, as we compared sequences of different lengths we observed a shift from primacy for short lists to recency for longer lists (Fig. 9).

**Fig. 10:**
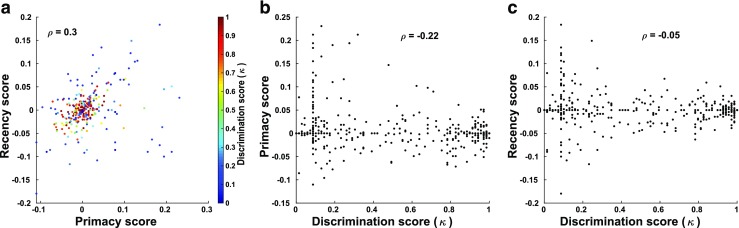
Relationships between recall accuracy measures of primacy, recency, and discrimination ability for the multi-item task. **a** Scatterplot of primacy *vs.* recency scores. Color indicates discrimination ability (***κ***). Primacy and recency scores were found to have a significant positive correlation (Pearson’s correlation: ***ρ = .*3**, ***p ≪ .*001**). **b** Scatterplot of primacy scores *vs.* discrimination ability. Primacy scores and ***κ*** values were found to have a significant negative correlation (Pearson’s correlation: ***ρ =  − .*22**, ***p ≪ .*001**) **c** Scatterplot of recency scores *vs.* discrimination ability. Recency scores and ***κ*** values were not found to be significantly correlated (Pearson’s correlation: ***p = .*067**). Insets: correlation coefficients between each pair of variables. Note that the majority of the data points in all 3 plots are overlapping and indistinguishable. In the left plot these overlapping points are at (0,0) while in the middle/right plots they are at (0,1)

We also measured primacy and recency in the scores of recall accuracy, by assessing how well the final state of the network could be used to identify the item at a given position in the sequence. Networks with the highest discrimination abilities exhibit neither primacy nor recency (Fig. 10). This is understandable, as a perfect network produces recall accuracy of 1, uniformly, across all list positions. However, networks in a large fraction of parameter space surrounding the optimal value do exhibit both primacy and recency, while still maintaining discrimination ability above 60%. It is possible to find networks exhibiting significant primacy and recency, but their discrimination ability is significantly lower. The lack of both primacy and recency for high-performing networks was one cause of a significant positive correlation between the amount of primacy and recency across networks (Fig. 10a) and a significant negative correlation between the amount of primacy and discrimination ability (Fig. 10b), although the latter trend was not seen for the effect of discrimination ability on recency (Fig. 10c).

### Network dynamics

Our prior work on randomly-connected attractor networks with synaptic depression (Miller 2013) established that these networks are capable of encoding stimulus features such as amplitude, duration, and number in a high-dimensional activity space. These three features are conflated by perfect integrators whose response is only the result of the product of these quantities. We showed through simulations that the key to the network’s stimulus discrimination abilities is the inclusion of depressing synapses. Synaptic depression of the excitatory self-synapses renders a unit less excitable and less likely to stay on upon subsequent external stimulus. In that task, successive stimuli in any sequence were identical, so synaptic depression was essential to move the activity pattern to new units. However, when the stimuli themselves activate different units, as in the tasks we analyze here, it is possible that synaptic depression is not required.

Therefore, we tested the importance of synaptic depression in the two tasks studied in this work, with six stimuli of two types, or with seven stimuli of seven types. For neither task were we able to find a set of parameters where a network retained any information of multiple stimuli in the sequence, so no networks were categorized as performing well. In the task with just two types of stimulus, repetitions of stimuli always occur, and in order for a repeated stimulus to produce a new network state, just as we found in our prior work, synaptic depression is essential. In the seven-item task, we expected to find networks in which successive stimuli produced new states, but we were unable to find any cases in which new states were history-dependent and task performance was minimal (data not shown).

**Fig. 11:**
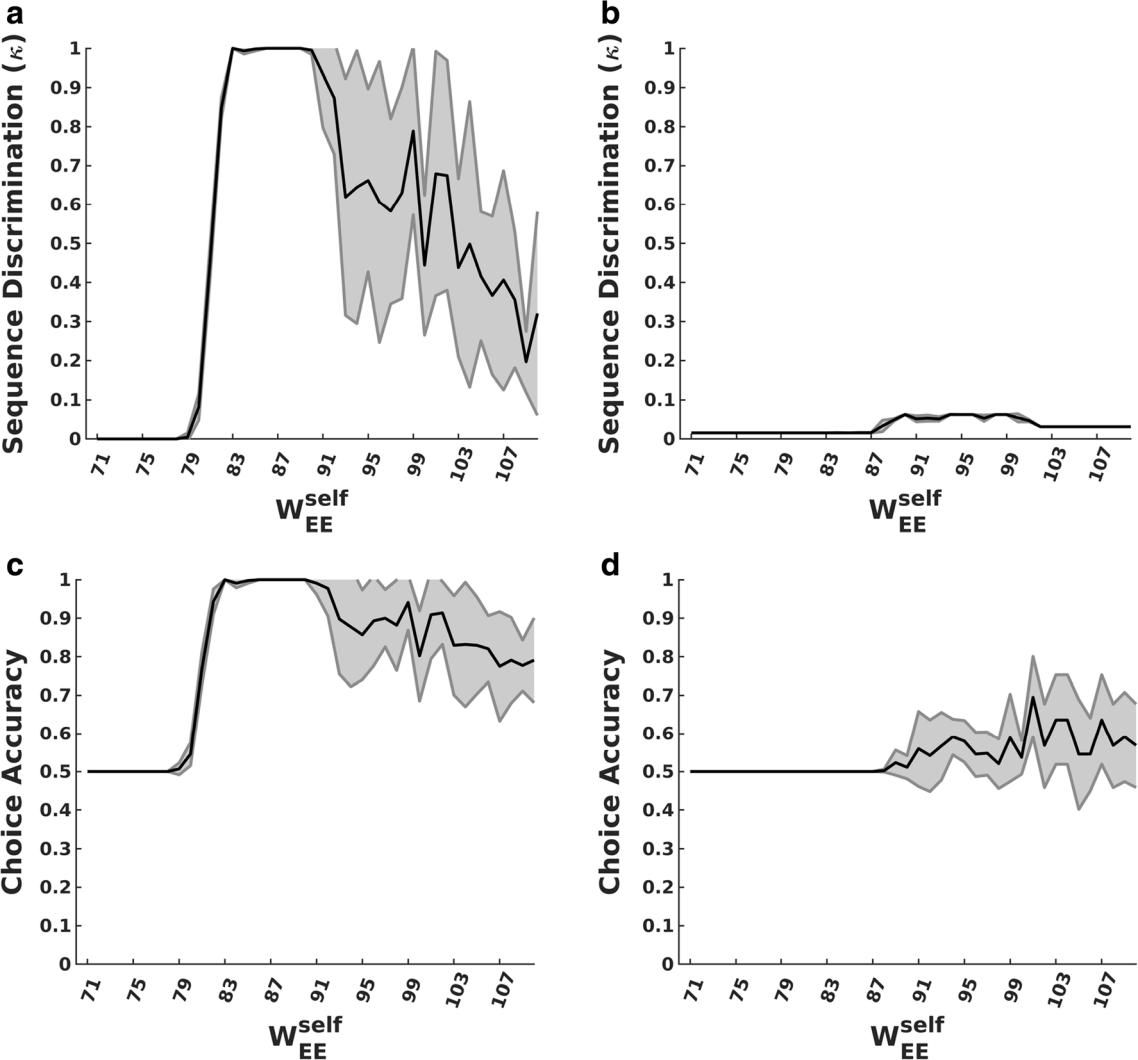
Effect of removing excitatory cross-connections. **a–b** Sequence discrimination (***κ***) with (**a**) and without (**b**) excitatory cross-connections as a function of excitatory self-connection strength. **c–d** Choice accuracy in the 2-choice task with (**c**) and without (**d**) excitatory cross-connections as a function of excitatory self-connection strength

While the excitatory self-connections produce the bistability within units that is necessary for history dependence, it is not so clear that excitatory connections between units are also vital for good task performance. We therefore tested networks without such cross-connections and found very limited performance (Fig. 11), demonstrating that the cross-connections are also vital to the network’s discrimination abilities. Such random excitatory cross-connections add to the heterogeneity of the network and help to preferentially “prime” new units in a history-dependent manner, such that the set of next units to switch on depends on the set of prior active units.

## Discussion

Our brains allow us to learn to respond appropriately to sequences of events rather than to be merely reactive to each ongoing stimulus. Therefore, circuits of neurons which can produce activity patterns containing information about the temporal pattern of prior stimuli have been an active area of research in computational neuroscience. Particularly powerful have been networks derived from liquid state machines (Maass et al. 2002; Jaeger et al. 2007), in which a stimulus produces a temporary, decaying trace of neural activity, but which interacts with later stimuli and can be extracted by training an appropriate “readout”. More recent work has shown how connections within such circuits can be trained to enable the network to learn almost any stimulus-response mapping (Sussillo and Abbott 2009; Barak et al. 2013; Nicola and Clopath 2017), even in a manner that is robust to noise and time-warping (Laje and Buonomano 2013; Goudar and Buonomano 2018).

Our work also follows the paradigm of the neocortex containing neural circuits that can serve as general-purpose computational machines. When the neural activity produces high-dimensional discrete attractor states, which are inherently robust and stable, an appropriate output can be readily trained through reinforcement learning, especially when just a binary response is necessary (Bourjaily and Miller 2011, 2012; Soltani and Wang 2008, 2010; Seung 2003). Therefore, while point-attractor networks lack the dynamic versatility of those based on chaotic systems, so would be less suitable as a basis for motor output, they may be more easily trained for complex cognitive tasks (Rigotti et al. 2010a, b), which inherently require short-term memory (Ganguli et al. 2008a, b). However, in the point-attractor circuits analyzed in this paper, the information pertaining to the history of a sequence of stimuli is present in the network **before training.** The training is only necessary to produce the correct categorization or behavioral response given pre-existing network information.

### First recall and overall recall

The first item recalled following a list of stimuli must be generated by the activity in the network that remains after presentation of all stimuli. While one may think that the most recent item would always have the strongest effect on the final activity state, there are broad regions of parameter space where the first item imprints certain activity patterns that are hard to shift. Moreover, the first stimulus is privileged, having a greater impact on network activity, if pre-stimulus activity is sparse or silent. As the sequence of stimuli grows, more and more changes in the initial state accrue until the final network state becomes more like later than like the first state. Such behavior is observed with changing list lengths in human free recall data. However, in our simulations, in those cases in which long sequences left the final network state closest to that induced by the first stimulus, the network retained little information about late stimuli and was indeed in some cases not influenced by them. Thus, at face value, in 10-word lists when the first item recalled is the first word, then only a few of the other early items would be recalled and none of the later ones. Such behavior would be intriguing if extracted from the human behavioral data.

We found it rare for a network to exhibit both primacy and recency while performing well. We expect this is in part due to the absence of many important features in the randomly connected circuit. In particular, in this paper, which we use as an initial demonstration of the capabilities of a naive network, we do not include correlational synaptic strengthening. Such strengthening is undoubtedly important in linking together the representations of successively presented words to form the observed associations, which would aid their recall (Howard and Kahana 1999; Howard et al. 2007; Miller and Wingfield 2010). Such information, which lies outside of the final state of activity in the network as used in our assessments here, is most likely essential for list recall in the multiple-item presentation task. Changes in protocols which impact the formation of such associations—for example, by varying time intervals, or incorporating distractors (Kahana 2012)—are known to impact the patterns of word recall, in a manner that cannot be reproduced in our network while it lacks synaptic plasticity.

### Impact of noise

Even in the absence of noise, the networks’ ability to discriminate between sequences of six or seven stimuli with minimal variations in the sequences is non-trivial. While distinct stimuli cause trajectories to diverge in chaotic networks, the history-dependence of the trajectory of activity in attractor networks has received less widespread attention. Whereas chaotic networks are naturally extremely sensitive to noise fluctuations, so careful attention to their robustness to such fluctuations can be necessary when training them to solve tasks, point attractor states are inherently robust to noise fluctuations up to the size of the barrier between attractor states. In our network the size of the barrier corresponds to a fluctuation in firing rate sufficient to render an active unit quiescent or a quiescent unit active. Therefore, we investigated the robustness of sequence discrimination to noise fluctuations in our networks.

In the point-attractor networks, the activity states remain relatively stable between stimuli and noise has its strongest impact during a stimulus when the system switches from one state to one of the many other states nearby in high-dimensional space. At such switching times, the network is much more sensitive to noise fluctuations as the barriers separating the zero-noise trajectory from trajectories to nearby attractor states is much smaller than the barriers between the attractor states themselves. Yet, even without training (and alterations of connection strengths), we did find that some networks were capable of producing good performance—at least at the human level—even while exhibiting considerable variability in their firing rates (e. g. 2–3 Hz fluctuations during the delay periods).

### Experimental predictions

**Fig. 12:**
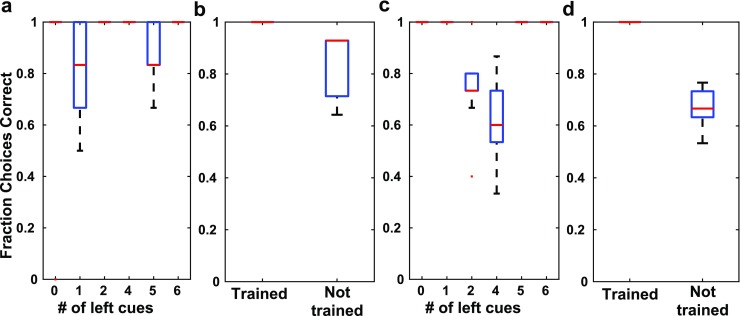
Choice accuracy on trained *vs.* not trained sequences in the 2-choice task. Data shown are from 10 networks generated using the same parameter set. For all boxplots, red lines indicate the median, blue boxes indicate the range from 25th to 75th percentile, whiskers extend to 3 Median absolute deviations (MADs), and red “+” signs mark outliers (defined to be points beyond 3 MADs from the median. **a–b** Perceptrons were trained only on sequences where there were 4 of one stimulus type in the sequence (or equivalently 2 of the other stimulus type). (**a**) shows the distribution of choice accuracy (across the 10 networks) for sequences where there is more of one stimulus type than the other. (**b**) shows mean fraction of correct choices for sequences that were used to train the perceptrons or not. **c–d** Perceptrons were trained only on sequences where there were 5 or 6 of one stimulus type in the sequence (or equivalently 0 or 1 of the other stimulus type). (**c**) as in (**a**) shows mean error rates for sequences where there is more of one stimulus type than the other. (**d**) as in (**b**) shows the mean fraction of correct choices for sequences that were used to train the perceptrons (left) or not (right)

In the 2-choice task, our model network is able to achieve correct choices by first discriminating all sequences from one another, regardless of the number of each stimulus in the sequence, and then mapping the resulting network states to correct choices through training. This process is different than the abstract notion of counting done by perfect integrators and makes testable predictions regarding neural activity and the behavior it produces. First, to determine whether mice in the T-maze task are mapping unique stimulus sequences to outcomes or using an abstract notion of a count, they could be trained on the task using sequences that contain only 2 or 4 left stimuli (*e.g.* LLRLRL, RRLLRR, *etc.*). If the animal is indeed using a counting strategy, then it should be able to generalize well and make correct choices when presented with sequences that contain 0, 1, 5, or 6 left stimuli (*e.g.* LLLLLL, RRRRLR, *etc.*). However, if the animal is mapping specific sequences to choice outcomes, as our network does, then choice accuracy should be worse on sequences that are not trained (Fig. 12).

The specificity of input patterns is one example of many in which our network is unable to generalize from training data to new data. The network’s ability to discriminate different qualities of the stimuli (as in Miller 2013) run counter to its ability to generalize its response when trained with one stimulus type to a test with a new stimulus type. This suggests that if stimulus features (such as their intensity or duration) were significantly changed in a test trial, then performance would worsen (as in Supplementary Figure 2). However, if the animal were trained on a diversity of stimulus types, it could regain its high performance (as in Supplementary Figure 4).

**Fig. 13:**
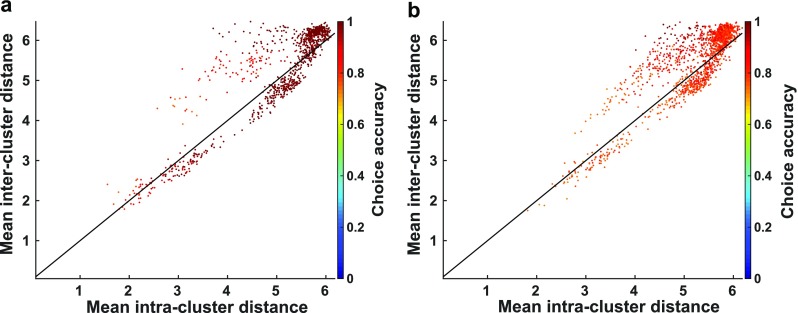
Separation of network states resulting from sequences with same number of “left” stimuli is similar to the separation of states resulting from sequences with different numbers of “left” stimuli. **a** Scatterplot of mean intra *vs.* inter-cluster distances for all networks in Fig. 4a that achieved a choice accuracy of > .73. A “cluster” is defined to be the set of binarized final network states produced by all sequences containing the same number of “left” (or “right”) stimuli. The mean inter-cluster distance across networks was found to be slightly but significantly greater than the mean intra-cluster distance (two-sample t-test: ***p ≪ .*001**). **b** Format is the same as **(a)** but shows data from all networks in Fig. 4b (noise value ***σ = .*002**) that achieved a choice accuracy > .73. Again, the mean inter-cluster distance across networks was found to be slightly but significantly higher (two-sample t-test: ***p ≪ .*001**). (Network states are defined as a binary vector, where each unit is designated to be “on” or “off” depending on whether its stable firing rate following a sequence of stimuli is greater than 30 Hz)

Further evidence that networks, such as the posterior parietal cortex, involved in processing stimulus sequences may utilize many discrete attractor states to encode sequences could be extracted by looking at how neural activity resulting from sequences with the same or different number of “left” stimuli cluster in activity space. In networks that performed well, we in general find that activity states produced by sequences with the same number of left stimuli are about as tightly (or loosely) clustered (as measured by Euclidean distances between pairs of activity states) as those with a different number of “left” stimuli (Fig. 13). Such neural behavior could be verified *in vivo* by measuring distances between the final network states produced by sequences with different numbers of each component stimulus type.

## Electronic supplementary material

Supplementary Figure 1Robust sequence discrimination is possible without an initially quiescent network. A-D show sequence discrimination scores (***κ***) as a function of self and cross-excitatory connection strengths. In each of A-D, a different pair of parameters $\Big({\boldsymbol{f}}_{\boldsymbol{start}}^{\boldsymbol{on}}$ and ***f***_***stim***_) were used. A) ${\boldsymbol{f}}_{\boldsymbol{start}}^{\boldsymbol{on}}=.\mathbf{2}$ and ***f***_***stim***_***= .*59** (273 evenly spaced parameter pairs were sampled) B) ${\boldsymbol{f}}_{\boldsymbol{start}}^{\boldsymbol{on}}=.\mathbf{59}$ and ***f***_***stim***_***= .*59** (36 evenly spaced parameter pairs were sampled) C) ${\boldsymbol{f}}_{\boldsymbol{start}}^{\boldsymbol{on}}=.\mathbf{59}$ and ***f***_***stim***_***= .*2** (36 evenly spaced parameter pairs were sampled) D) ${\boldsymbol{f}}_{\boldsymbol{start}}^{\boldsymbol{on}}=.\mathbf{2}$ and ***f***_***stim***_***= .*2** (36 evenly spaced parameter pairs were sampled) (PNG 1462 kb)

High Resolution (TIFF 140634 kb)

Supplementary Figure 2 Sequence discrimination scores resulting from variable stimulus durations or amplitudes. A-D show sequence discrimination scores as a function of cross-inhibition weights ***W***_***EI***_ and ***W***_***IE***_. A) Sequence discrimination scores in networks with constant stimulus amplitude and duration and no noise (***σ =*0**). 36 evenly spaced parameter pairs were sampled. B) Sequence discrimination scores in networks with constant stimulus amplitude and duration with significant noise (***σ = .*002**). 121 evenly spaced parameter pairs were sampled. C) Sequence discrimination in networks with variable stimulus amplitude (stimulus amplitudes were chosen from a Gaussian distribution with mean ***I***_***stim***_ and standard deviation ***.*1*∗ I***_***stim***_. 36 evenly spaced parameter pairs were sampled. D) Sequence discrimination scores for networks with variable stimulus duration. Stimulus durations were chose from a Gaussian distribution with mean ***T***_***stim***_ and standard deviation ***.*1*∗ T***_***stim***_. 143 evenly spaced parameter pairs were sampled. (PNG 8710 kb)

High Resolution (TIFF 140634 kb)

Supplementary Figure 3Sequence discrimination and choice accuracy (2-choice task) scores when the decoder/perceptron was trained on sequences with an inter-stimulus-interval of 1500ms while being tested on sequences with an inter-stimulus-interval of 3000ms. A) Sequence discrimination (***κ***) as a function of self and cross-excitation strengths. B) Choice accuracy as a function of self and cross-excitation strengths. 36 evenly spaced parameter pairs in the $\left({W}_{\mathrm{EE}}^{\mathrm{self}},{W}_{\mathrm{EE}}^{\mathrm{max}}\right)$ space were sampled (PNG 4705 kb)

High Resolution (TIFF 140634 kb)

Supplementary Figure 4Generalization of choice accuracy to sequences with different stimulus amplitudes. All networks were presented with 128 training and 128 test sequences. Half of both the training and test sequences had stimulus amplitude ***I***_***stim***_and half had amplitude ***I***_***stim***_***+ .*1*∗ I***_***stim***_. a) Mean sequence discrimination (***κ***) for 25 evenly spaced parameter sets interpolated over the space of self and cross-excitation weights. b) Same as (a) but plotting 2-choice accuracy. 36 evenly spaced parameter pairs in the $\left({W}_{\mathrm{EE}}^{\mathrm{self}},{W}_{\mathrm{EE}}^{\mathrm{max}}\right)$ space were sampled (PNG 4226 kb)

High Resolution (TIFF 140634 kb)

Supplementary Figure 5Discrimination ability in the 7-word multi-item task for a range of networks in the self/cross-excitation space. a) Networks with no noise (***σ =*0**). 121 evenly spaced parameter pairs in the $\left({W}_{\mathrm{EE}}^{\mathrm{self}},{W}_{\mathrm{EE}}^{\mathrm{max}}\right)$ were sampled. b) Networks with moderate noise (***σ = .*002**). 25 evenly spaced parameter pairs in the $\left({W}_{\mathrm{EE}}^{\mathrm{self}},{W}_{\mathrm{EE}}^{\mathrm{max}}\right)$ space were sampled (PNG 5022 kb)

High Resolution (TIFF 140634 kb)

ESM 1 3-dimensional view of the network activity from Fig 8a. Principal components analysis was carried out on the network activity in response to 2 sequences that differ only in their first stimulus (“010101” vs. “110101”) to define the axes. The red dot indicates the current activity state due to the top sequence in Fig8a (“010101”) while the tail shows the activity over the preceding 400ms. Similarly, the blue dot indicates the current activity due to the bottom sequence in Fig8a (“110101”) while the tail shows the activity over the preceding 400ms. The numbers that appear indicate the stimulus number and mark the respective activity states at the beginning of the stimulus (MOV 7077 kb)

ESM 2 3-dimensional view of the network activity from Fig 8b. Principal components analysis was carried out on the network activity in response to 2 sequences that differ only in their third stimulus (“010101” vs. “011101”) to define the axes. The red dot indicates the current activity state due to the top sequence in Fig8b (“010101”) while the tail shows the activity over the preceding 400ms. Similarly, the blue dot indicates the current activity due to the bottom sequence in Fig8b (“011101”) while the tail shows the activity over the preceding 400ms. The numbers that appear indicate the stimulus number and mark the respective activity states at the beginning of the stimulus (MOV 7125 kb)

ESM 3 3-dimensional view of the network activity in response to the presentation of 2 sequences of 6 different stimuli (“123456” vs. “723456”) that differ only in their first position. Principal components analysis was carried out on the network activity to define the axes. The red dot indicates the current activity state due to the sequence “123456” while the blue dot indicates the current activity due to the sequence “723456”. The blue and red tails indicate the respective activity over the preceding 400ms. The numbers that appear indicate the stimulus number and mark the respective activity states at the beginning of the stimulus (MOV 7202 kb)

ESM 4 3-dimensional view of the network activity in response to the presentation of 2 sequences of 6 different stimuli (“123456” vs. “127456”) that differ only in their first position. Principal components analysis was carried out on the network activity to define the axes. The red dot indicates the current activity state due to the sequence “123456” while the blue dot indicates the current activity due to the sequence “127456”. The blue and red tails indicate the respective activity over the preceding 400ms. The numbers that appear indicate the stimulus number and mark the respective activity states at the beginning of the stimulus (MOV 7138 kb)
