# Supplementary figures and images for: Spatiotemporal discrimination in attractor networks with short-term synaptic plasticity
Source: J Comput Neurosci. 2019 May 27;46(3):279–97. doi: 10.1007/s10827-019-00717-5 (PMC6571095; doi:10.1007/s10827-019-00717-5)

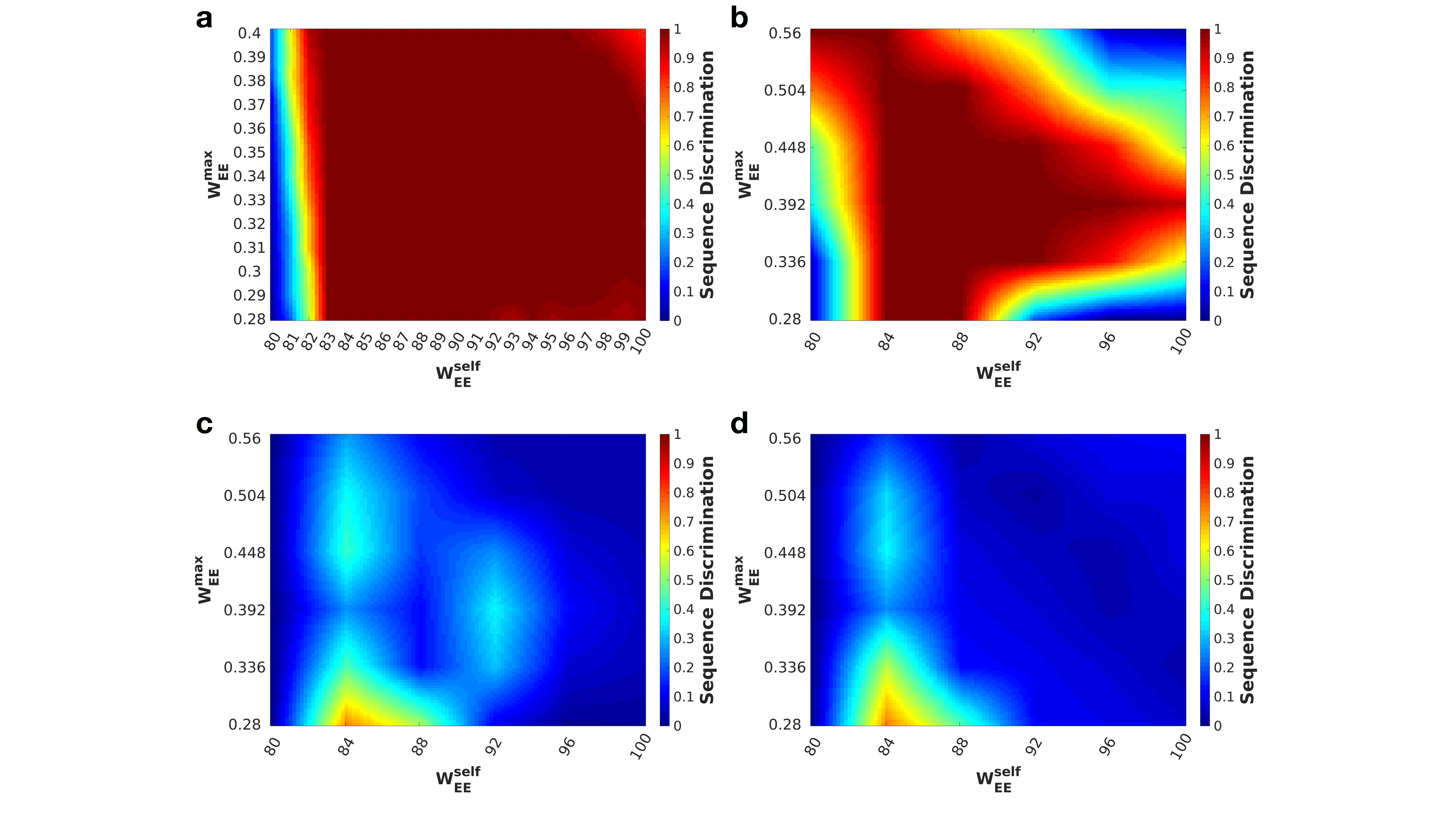

Supplement: Supplementary file 1 — Robust sequence discrimination is possible without an initially quiescent network. A-D show sequence discrimination scores (κ) as a function of self and cross-excitatory connection strengths. In each of A-D, a different pair of parameters $\Big({\boldsymbol{f}}_{\boldsymbol{start}}^{\boldsymbol{on}}$(fstarton and fstim) were used. A) ${\boldsymbol{f}}_{\boldsymbol{start}}^{\boldsymbol{on}}=.\mathbf{2}$fstarton=.2 and fstim= .59 (273 evenly spaced parameter pairs were sampled) B) ${\boldsymbol{f}}_{\boldsymbol{start}}^{\boldsymbol{on}}=.\mathbf{59}$fstarton=.59 and fstim= .59 (36 evenly spaced parameter pairs were sampled) C) ${\boldsymbol{f}}_{\boldsymbol{start}}^{\boldsymbol{on}}=.\mathbf{59}$fstarton=.59 and fstim= .2 (36 evenly spaced parameter pairs were sampled) D) [file 10827_2019_717_Fig14_ESM.png]
